# Quantification of spatial metal accumulation patterns in *Noccaea caerulescens* by X-ray fluorescence image processing for genetic studies

**DOI:** 10.1186/s13007-021-00784-9

**Published:** 2021-08-03

**Authors:** Lucas van der Zee, Amelia Corzo Remigio, Lachlan W. Casey, Imam Purwadi, Jitpanu Yamjabok, Antony van der Ent, Gert Kootstra, Mark G. M. Aarts

**Affiliations:** 1 Farm Technology, Department of Plant Sciences, Wageningen University and Research, Wageningen, The Netherlands; 2 Laboratory of Genetics, Department of Plant Sciences, Wageningen University and Research, Wageningen, The Netherlands; 3 Centre for Mined Land Rehabilitation, Sustainable Minerals Institute, The University of Queensland, Brisbane, Australia; 4 Centre for Microscopy and Microanalysis, The University of Queensland, Brisbane, Australia

**Keywords:** Metal hyperaccumulation, µXRF, Imaging, Image segmentation, *Noccaea caerulescens*, Heritability

## Abstract

**Background:**

Hyperaccumulation of trace elements is a rare trait among plants which is being investigated to advance our understanding of the regulation of metal accumulation and applications in phytotechnologies. *Noccaea caerulescens* (Brassicaceae) is an intensively studied hyperaccumulator model plant capable of attaining extremely high tissue concentrations of zinc and nickel with substantial genetic variation at the population-level. Micro-X-ray Fluorescence spectroscopy (µXRF) mapping is a sensitive high-resolution technique to obtain information of the spatial distribution of the plant metallome in hydrated samples. We used laboratory-based µXRF to characterize a collection of 86 genetically diverse *Noccaea caerulescens* accessions from across Europe. We developed an image-processing method to segment different plant substructures in the µXRF images. We introduced the concentration quotient (CQ) to quantify spatial patterns of metal accumulation and linked that to genetic variation.

**Results:**

Image processing resulted in automated segmentation of µXRF plant images into petiole, leaf margin, leaf interveinal and leaf vasculature substructures. The harmonic means of recall and precision (F1 score) were 0.79, 0.80, 0.67, and 0.68, respectively. Spatial metal accumulation as determined by CQ is highly heritable in *Noccaea caerulescens* for all substructures, with broad-sense heritability (H^2^) ranging from 76 to 92%, and correlates only weakly with other heritable traits. Insertion of noise into the image segmentation algorithm barely decreases heritability scores of CQ for the segmented substructures, illustrating the robustness of the trait and the quantification method. Very low heritability was found for CQ if randomly generated substructures were compared, validating the approach.

**Conclusions:**

A strategy for segmenting µXRF images of *Noccaea caerulescens* is proposed and the concentration quotient is developed to provide a quantitative measure of metal accumulation pattern, which can be used to determine genetic variation for such pattern. The metric is robust to segmentation error and provides reliable H^2^ estimates. This strategy provides an avenue for quantifying XRF data for analysis of the genetics of metal distribution patterns in plants and the subsequent discovery of new genes that regulate metal homeostasis and sequestration in plants.

**Supplementary Information:**

The online version contains supplementary material available at 10.1186/s13007-021-00784-9.

## Background

Understanding the mechanism of metal accumulation in plants is of great importance. Large areas of the world are enriched with potentially toxic trace elements, either for anthropogenic reasons, such as metal mining and smelting, or for natural, geochemical reasons. Increased exposure of humans to certain metals affects human health, including an increased risk of cancer [1–3]. Most metals that affect human health also have a negative effect on plants. There are, however, some plant species that are extremely tolerant to metal exposure and that hyperaccumulate certain trace elements to very high concentrations in their leaves. Hyper accumulating species can be used to extract certain metals and metalloids from the soil [4], either to clean the soil, a process called phytoremediation, or even to extract valuable metals from soils containing sub-economic concentrations without disrupting the topsoil in a process called phytomining [5, 6]. Hyperaccumulator plants also take up trace elements essential for human health, such as iron (Fe), zinc (Zn), manganese (Mn) or copper (Cu). By understanding the plant physiology and the underlying genetics of metal accumulation, cultivars can be bred that contain higher contents of these micronutrients in their edible parts, which will benefit their nutritional value. Such biofortified cultivars can help fight mineral malnutrition, one of the major causes of human mortality worldwide [7]. To unlock these applications of metal accumulation, an improved understanding of the underlying genetics is required.

*Noccaea caerulescens* is a particularly interesting species to study in this regard. It can accumulate and tolerate extraordinary concentrations of Zn, cadmium (Cd), and nickel (Ni) [8], and some accessions even lead (Pb). Moreover, there is substantial variation in metal accumulation genotype and phenotype between different populations of this species [8–10]. Several genes are known to participate in metal hyperaccumulation. These include genes for metal influx transporters that take up metals into the cytosol [11, 12], for metal tonoplast transporters, which transport metals and their chelators to and from the vacuole [13, 14], for metal efflux transporters, which exclude excess metals from the cytosol and are involved in transportation towards the shoot [12], and for a range of regulatory and signalling proteins [15]. Yet many genes remain to be uncovered to gain a full understanding of metal accumulation.

While total metal concentrations are routinely obtained using destructive sample analysis techniques, such as Inductively Coupled Plasma Atomic Emission Spectroscopy/Mass Spectrometry (ICP-AES/MS), more complex phenotypic traits remain underexplored, such as the spatial distribution of the metallome across plant tissues. Previous work on metal distribution was limited to mostly qualitative analysis. For instance, leaf tip Zn accumulation and a homogenous leaf distribution of Ni was found using laser ablation inductively coupled plasma mass spectroscopy [16]. Fluorescent probes were used in combination with microscopy to find that Zn accumulation is not affected by plant age, that phosphorous concentration decreases upon Zn accumulation in older plants and that Zn mainly locates to the apoplastic space of leaf epidermal cells [17]. Spatial accumulation patterns were found not to differ between plants grown in a natural environment versus those grown hydroponically [18]. Finally, the use of synchotron-based X-ray fluorescence microscopy on the closely related *Noccaea tymphaea* uncovered that Zn and Ni do not necessarily colocalize in *Noccaea* [19]. Overall, these studies lack the comparison of metal accumulation distribution phenotypes across many accessions. Image-processing and elemental mapping methods will enable discrimination between traits that are specific to the accessions and those that hold for the whole species. Also, it will allow for the determination of genetic heritability of metal distribution patterns which can lead to the discovery of novel genes and processes that regulate metal homeostasis.

X-ray Fluorescence Spectroscopy is a non-destructive elemental analysis technique that is capable of detecting a wide range of elements covering most of the plant metallome [20, 21]. Micro X-ray Fluorescence (µXRF) uses an X-ray source to illuminate a sample enabling mapping fresh or hydrated plant specimens with a spatial resolution as small as 1 µm and a detection limit as low as 5 µg g^−1^ for transition metals [22]. µXRF is singularly suited for such applications, as it is a non-destructive method for obtaining quantitative spatial information of elemental distribution in physically intact biological materials that avoids introducing artefacts associated with standard sample preparation protocols, such as sectioning and fixation [23].

**Fig. 1:**
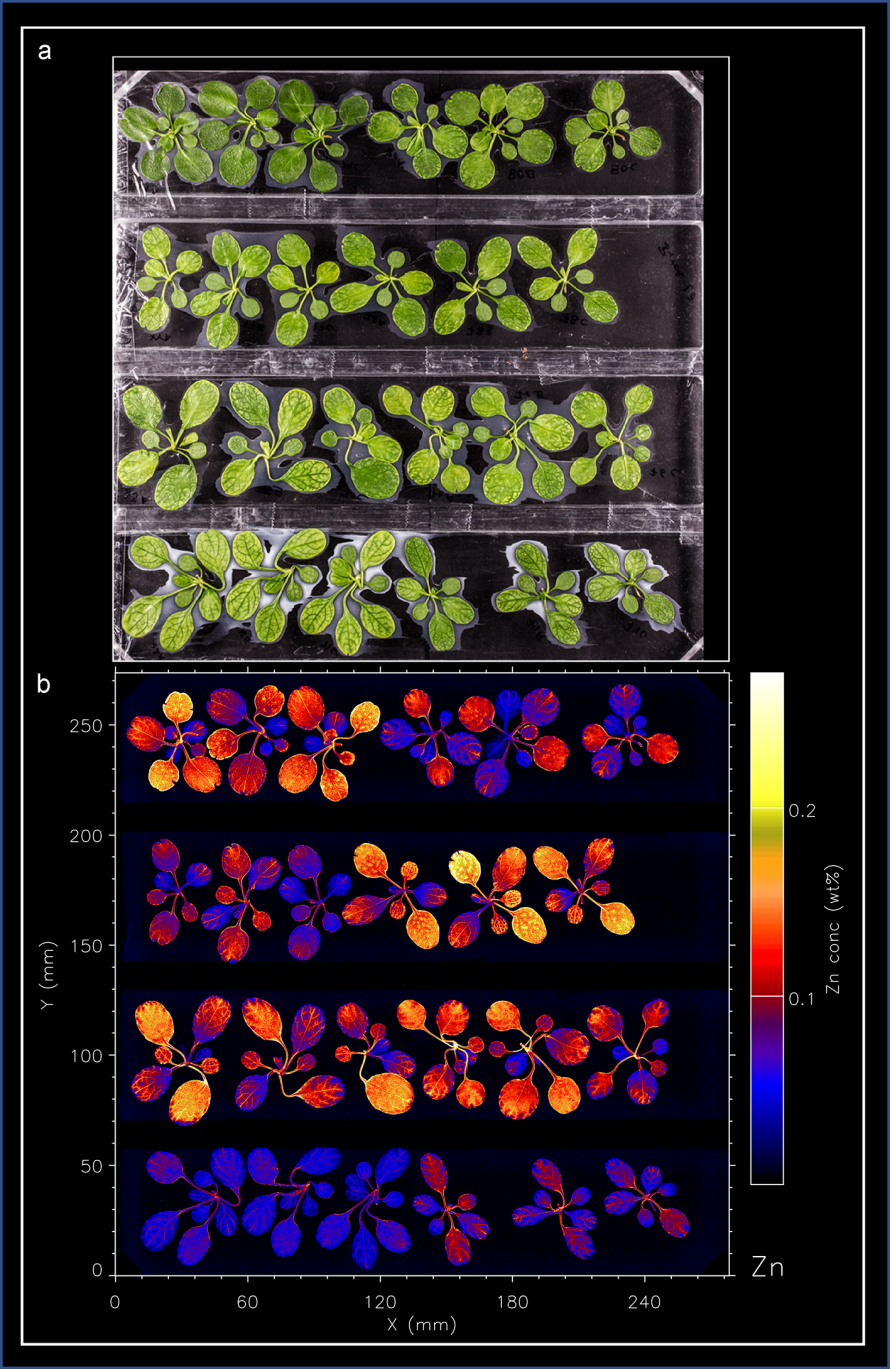
A batch of 24 *N. caerulescens* plants, corresponding to three plants of eight accessions, grouped together per accession, imaged by **a** photography, and **b** processing of the µXRF scan to visualize Zn concentration as a false-colour image, with black corresponding to the lowest and white to the highest Zn concentrations

When initially viewing µXRF scans of different accessions of *Noccaea caerulescens*, differences were observed in the spatial distribution of metals in leaves, the petiole, leaf margin, leaf vasculature and leaf interveinal substructures (Fig. 1). The petiole anatomically consists largely of vascular bundles and hence the XRF signal would be dominated by elements that concentrate in the phloem (given that xylem is typically dilute). Based on physiological knowledge, differences between petiole and leaf blade vascular bundles are therefore expected to be small. To test these qualitative observations, substructures must be segmented by classifying each pixel of the image as belonging to the background or one of the substructure classes. Image-processing techniques can segment images into regions representing these substructures by considering characteristics of their shape and/or pixel intensities. Every pixel in the image can be classified to belong to a certain substructure. A complete review of machine-vision algorithms for plant-part segmentation is out of the scope of this article, but some relevant examples are described below. Thresholding is often used to extract an object from a uniform background based on the pixel intensity or colour [24, 25] Instead of using a static threshold, dynamic thresholding techniques, such as Otsu thresholding or local thresholding, make the algorithm more robust to variations in illumination. A leaf can be subdivided in different substructures using classical image-processing techniques. For instance, morphological opening can be used to segment the petiole from the blade [26]. Leaf margins can be detected using the morphological gradient operation to mark an edge of arbitrary width along the foreground mask of the leaf [27]. Vasculature can be detected based on their small width using morphological opening [24, 28]. Leaf vasculature has also been separated from the leaf tissue based on pixel intensities [25]. Alternatively, edge-detection algorithms based on the second-order image derivative, such as the Laplacian operator, have been used to identify vasculature [29]. Lastly, ‘Artificial Ants’ have been used too to segment the vasculature by tracing edges [30, 31]. Active-contour methods form another set of image-processing techniques that allow the detection of plant parts [32, 33]. In recent years, deep-learning techniques have become popular for plant-part segmentation, e.g., [34–36]. These methods, however, require a lot of training data. For this study, a method based on classical image-processing techniques shows to be more effective. Our method uses intensity thresholding to segment the plant from the background. Blade and petiole are separated using morphological opening. The leaf margin is found through a morphological gradient operation, and finally, the leaf vasculature and interveinal tissue are separated using a Laplacian operator.

The objective of this study is to develop a method to quantify the spatial distribution of metal accumulation in different plant substructures and to determine the broad-sense heritability of these metal distribution phenotypes. To this end, we have spatially mapped the metallome by µXRF in a diversity panel of different *N. caerulescens* accessions grown in a hydroponic nutrient solution enriched in Zn and Ni. We propose an image-processing method to segment the µXRF images of *N. caerulescens* into four different substructures (petiole, leaf margin, leaf interveinal and leaf vasculature). Consecutively, we introduce a numerical description of spatial metal distribution, the concentration quotient (CQ), which is used to determine the heritability of spatial metal distributions across the *N. caerulescens* diversity panel. While data are obtained for the whole metallome, the analysis focuses exclusively on the two elements supplied in excess, Zn and Ni, and two general, abundant elements, K and Ca.

## Results

### Metal distribution in the plants

The *Noccaea caerulescens* accessions are grown in batches of eight accessions each, in hydroponics, with five plants per accession. The three best growing plants are used for µXRF analysis. The three plants per accession are highly comparable in plant size and appearance, between accessions there is considerable variation, reflecting the genetic variation that is expected for a diversity panel of accessions originating from the north-west of Spain to the south of Finland (see Additional file 1). After 1 week of hydroponic growth at 2 µM Zn and no Ni, plants are supplied with fresh solution containing 20 µM Zn and 100 µM Ni (all as sulfate salts), to induce accumulation of both Zn and Ni. Plants continue to grow well, although some accessions develop mild chlorosis of the leaf blades, but no signs of metal toxicity or severe deficiency are observed when shoots of three plants per accession are mounted for µXRF analysis (Fig. 1).

The possibility of radiation-induced damage in µXRF analysis (especially in fresh hydrated samples) is an important consideration that may limit the information sought from the analysis [23]. The aim of µXRF analysis is to examine physiologically as close as possible to the natural state of the plant. ‘Damage’ can be defined as any change to the specimen that compromises the examination of these processes. We could not discern any physiological changes (such as wilting, discoloration, etc.) consequential to the µXRF analysis. This can be explained because the source produces a flux of 2.2 × 10^8^ photons s^−1^ in a 25-μm beam spot, at a maximum dwell of 100 ms. This results in a deposited radiation dose of just 6.6 Gy [23]. Throughout the study, Zn and Ni will be considered, which are the metals provided at elevated concentrations, and which are known to be hyperaccumulated by *N. caerulescens*. In addition, two reference metals, Ca and K are considered, which are normally abundant and well spread over the leaf. Total plant metal content is calculated from the µXRF scans (Fig. 1 for Zn) and compared to ICP-AES data obtained from material collected after the µXRF-scanning (see Additional file 2: Fig S1). These two datasets correlate reasonably well for all metals (Pearson’s *r* = 0.87, 0.83, 0.78, 0.73 for Zn, Ca, K, and Ni respectively).

### Segmentation quality

**Fig. 2:**
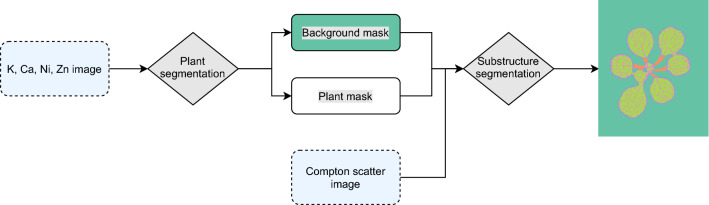
Flow chart of the segmentation algorithm. Blue striped boxes denote inputs, grey diamonds denote image processing steps. White box denotes an intermediate result, colours denote the five output classes

**Fig. 3:**
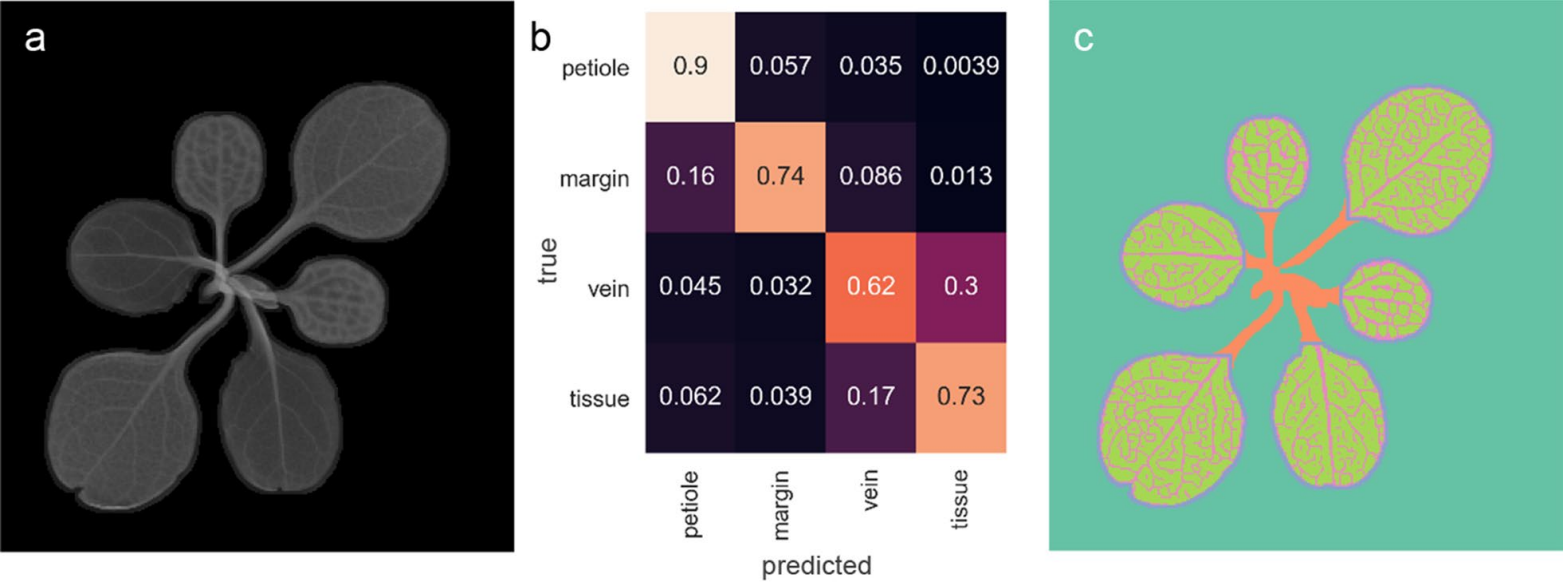
Comparison of Compton scatter image and pixel-for-pixel classification. **a** Compton scatter X-ray image of a *Noccaea caerulescens* plant. **b** row-normalized confusion matrix showing evaluation of ground-truth. Horizontal axis sums up to 1. **c** Classification of the major plant structures: petiole (orange), leaf margin (violet), leaf vasculature (vein)(pink) and leaf interveinal tissue (tissue)(green)

Using traditional image-processing techniques, a segmentation algorithm has been designed for the raw images which is applied to the dataset (Fig. 2). Four classes of plant substructures are discriminated: petiole, leaf margin, leaf vasculature (vein) and leaf interveinal tissue (tissue) (Fig. 3c). The algorithmic segmentation is evaluated using 4000 manually classified pixels. Considerable differences in recall and precision are seen between the classes (Fig. 3b). The petiole can be segmented with a recall of 90%. The recall for leaf margin is 74%, with 16% of the leaf margin pixels being erroneously predicted as petiole. Confusion also exists between vasculature and interveinal tissue pixels resulting in a recall for the vasculature class of 62% and for the tissue class of 73% (Fig. 3b). Precision scores for petiole, margin, vasculature, and tissue are, respectively, 0.696, 0.868, 0.721, and 0.633.

**Fig. 4:**
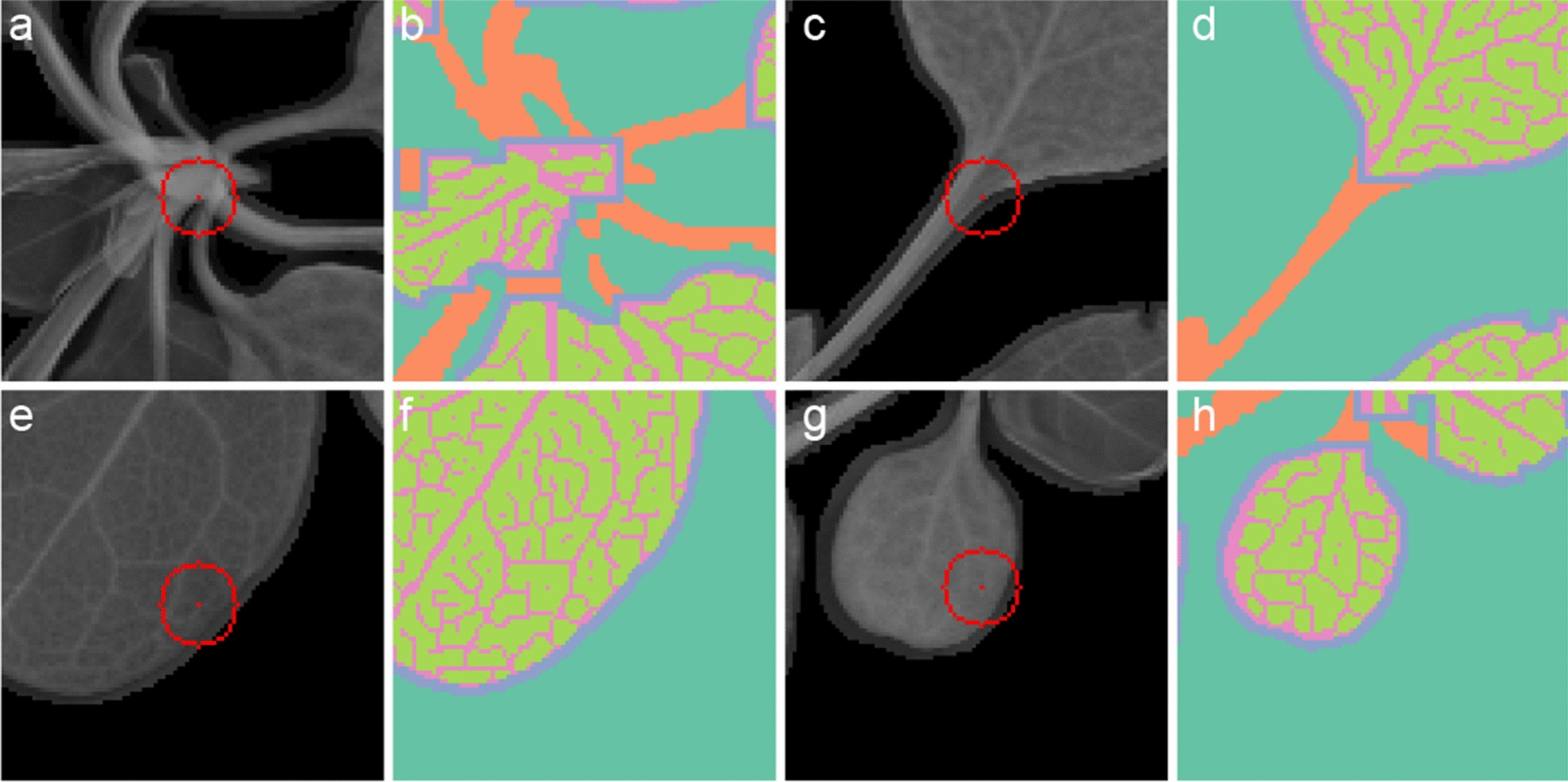
Examples of misclassified pixels. Compton scatter and segmentation of petiole (**a**, **b**), leaf margin (**c**, **d**), leaf vasculature (**e**, **f**), and leaf interveinal tissue (**g**, **h**)

Petioles are misclassified when the petiole crosses or closely borders a leaf. This is often the case for young developing leaves positioned close to the centre of the rosette (Fig. 4a, b). Margin pixels are misclassified as petiole when the leaf extends along the petiole (Fig. 4c, d), or wherever small developing leaves are erroneously classified as petiole. Vasculature and interveinal tissue are mixed up in the vicinity of tertiary vasculature because image resolution is too low to discriminate these very fine structures (Fig. 4e, f, g, h). Finally, the method cannot handle very small plants, which are completely mis-classified as “petiole”, but this only applied to three plants in the experiment.

**Fig. 5:**
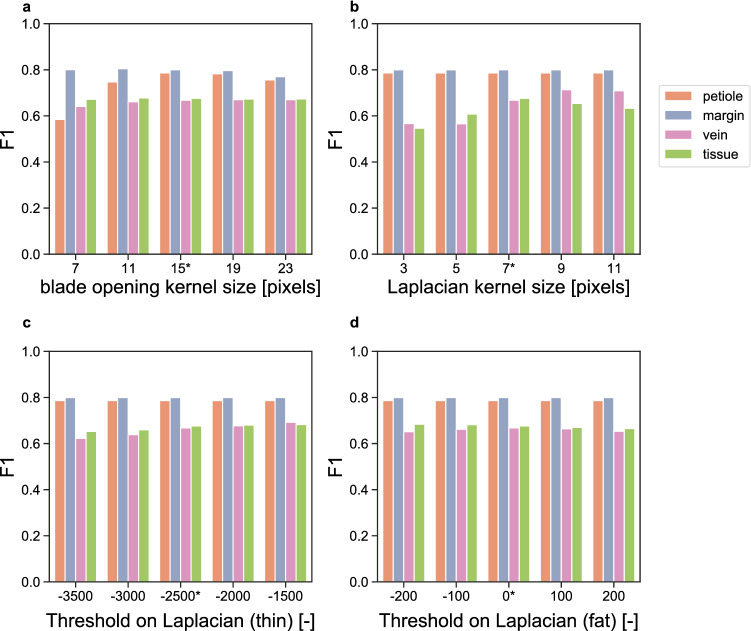
Sensitivity segmentation quality to four segmentation parameters by F1-score. **a** Kernel size used for morphological opening operation on binary plant masks to yield blade and petiole separation. **b** Kernel size of Laplacian operator to detect edges. **c** Binary threshold on the calculated Laplacian operator to segment thinnest vasculature. **d** Binary threshold on the calculated Laplacian operator to segment wider vasculature. Values on the X-axis with asterisk are used for all analyses. Substructures are indicated in orange (petiole), violet (leaf margin), lilac (leaf vasculature; vein) and green (interveinal tissue; tissue)

A sensitivity analysis shows effects of segmentation parameter values on the harmonic mean of precision and recall (F1-score). Increased F1-scores in one substructure often coincide with decreased scores in other substructures (Fig. 5). Increasing the kernel size used for the morphological opening causes a larger part of the plant to be classified as petiole (Fig. 5a). The increase in petiole F1-score coincides with decreased F1-scores for all other substructures. Sensitivity of the F1-score is tested for three parameters that influence vein and tissue segmentation; the Laplacian operator kernel size (Fig. 5b) and two binary thresholds that convert the result of the Laplacian operation into vasculature- and intervascular tissue masks (Fig. 5c, d). Increasing the kernel size causes more pixels to be classified as vein and less as tissue (Fig. 5b). This raises the F1-score for both vein as well as tissue. A similar pattern is visible for the threshold on the Laplacian that segments thin vasculature from tissue (Fig. 5c). Hardly any effect on the F1-score is observed when changing the threshold for segmenting thick vasculature (Fig. 5d); increases in recall are set off by decreases in precision and vice versa (see Additional file 2: Fig S2).

### Comparison of leaf substructures

**Fig. 6:**
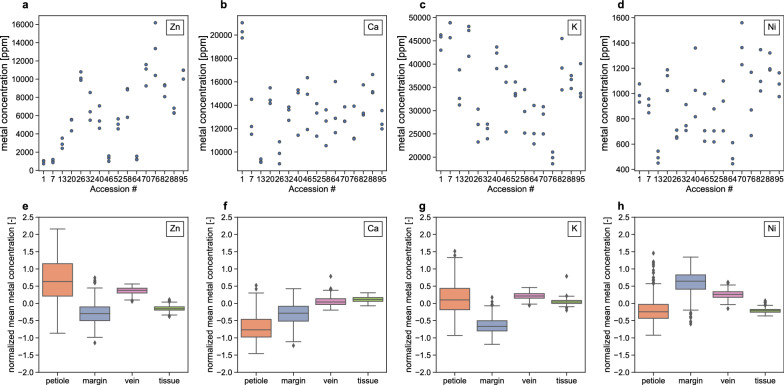
Distribution of shoot metal concentration for every 6th accession for respectively Zn, Ca, K, and Ni (**a**–**d**) as measured by ICP-AES. Comparison of normalized mean metal concentrations per substructure for all *N. caerulescens* accessions for respectively Zn, Ca, K, Ni, (**e**–**h**) derived from μXRF image analysis. Mean substructure metal concentrations are Z-score-normalized using the plant mean and standard deviation

To compare metal accumulation in substructures across all accessions, the metal concentrations are first normalized to the plant mean (Fig. 6). A large range of normalized mean substructure concentrations is visible for the petiole and margin across all metals. Remarkably, while Zn and Ni colocalize in most plants, this does not result in equal amounts of under- and overaccumulation of the two metals in any given substructure. The vasculature is the only substructure that consistently accumulates all metals above the plant mean. Other substructures over accumulate some metals but under accumulate others. The differences reported here are only observed when metal concentrations are normalized; the large variation in mean plant metal concentration between the accessions overshadows the differences of within-plant metal distribution (Fig. 6).

### Broad-sense heritability determination

**Fig. 7:**
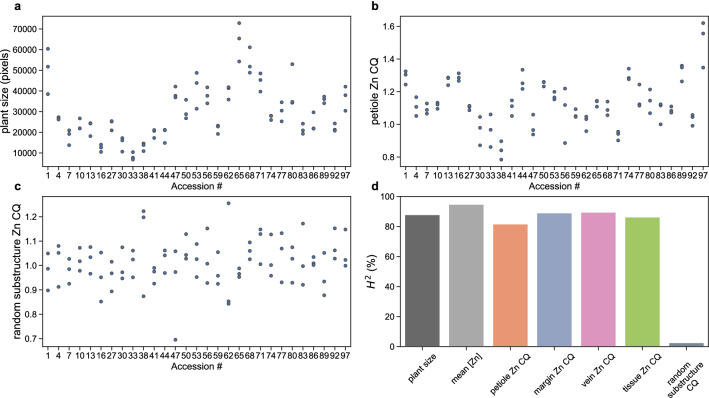
Genetic variation for traits determined based on the µXRF images for three plants per *N. caerulescens* accession, for **a** plant size, **b** zinc CQ values for the petiole, **c** zinc CQ values for a randomly drawn substructure. Every third accession in the dataset was chosen for display. In **d**, some of the broad-sense heritability (H^2^) scores (in % of total phenotypic variance) for indicated traits and for the random substructure CQ value are shown

Broad-sense heritability (H^2^) values indicate how much the phenotypic variance of a population can be explained by genetic rather than environmental factors [37]. H^2^ can be used to relate phenotypic traits to genetic variation and is an important tool for evolutionary biology and plant breeding [37, 38]. The higher the H^2^, the easier it will be to use the trait values for genetic analysis, as the phenotypic variation effectively reflects genotypic variation. Since we have determined phenotypes for three replicate plants of each genotypically homogeneous accession, we can estimate the non-genotypic (environmental/error) component of the total phenotypic variance, from the variance within genotypes. Next to several metal concentration and distribution traits, we have also measured plant size, which is for many plant species a trait with high heritability. As an example (Fig. 7), the phenotypic variation for plant size is high, when comparing the different accessions, but much smaller between plants of the same accession. Consequently, the broad-sense heritability of plant size is high, at 87.8% (Fig. 7d).

To determine the heritability of metal accumulation in substructures, a metric is sought that holds information on the accumulation in a substructure independent of the substructure’s size and mean concentration in the plant. The concentration quotient fulfils these demands. It expresses the mean concentration in the substructure divided by the mean concentration in the plant (Eq. 13). Similar to plant size, the Zn CQ values of the petiole (Fig. 7b) also show low variation within accessions, and high variation between accessions. The same is true for the Zn CQ values of all other analysed substructures. The plant mean concentration of Zn results in the highest H^2^ at 94.7% (Fig. 7d).

To exclude the possibility that the CQ-metric measures some other heritable trait by proxy, we have examined whether the metal CQs of substructures correlate with plant size, mean metal concentration or the size of the substructure relative to that of the plant. Metal CQs of the petiole, leaf margin and leaf interveinal tissue generally correlate only weakly with plant size and mean Zn, Ni, K, and Ca concentrations (see Additional file 2: Table S1). For Zn vein CQ there is some correlation with plant size (*r* = 0.15) and mean zinc concentration (*r* = 0.41). This indicates that plants with higher Zn concentrations tend to store it increasingly in their vasculature. Also, for the other three metals there is a correlation between metal vein CQ and mean concentration, but the correlation is not always positive (see Additional file 2: Table S1). Nickel vein CQ correlates positively with mean metal concentration (*r* = 0.38), while K- and Ca- CQ correlate negatively with mean metal concentration (respectively, *r* =  − 0.34, and* r* =  − 0.55). The latter means that K and Ca are not increasingly stored in the vasculature upon increasing metal concentration. All metals in all substructures correlate only weakly with the relative substructure size (see Additional file 2: Table S1).

As expected, there is negative pairwise correlation between the Zn CQs of the different substructures. Notably, the vein Zn CQ correlates to the margin Zn CQ with *r* = − 0.50 and margin Zn CQ correlates to petiole Zn CQ with *r* = − 0.41; an increased accumulation in one substructure is paired with a decreased accumulation in one or more other substructures. Similar correlations are found for pairwise substructure CQ correlations for other metals (See Additional file 2: Fig. S3).

As a control for the validity of CQ as a metric for metal accumulation heritability, random substructures are created for every plant. These random substructures consist of five square patches randomly distributed over the plant image. The H^2^ for the random substructures CQ is very low (Fig. 7c). Such low H^2^ is indeed expected unless there is an unforeseen problem with the CQ value calculation or the image processing, which is not the case.

Finally, we assessed the robustness of CQ values against classification errors, by introducing ‘noise’ into the classification algorithm. This is done by assigning a random class to a certain percentage of plant pixels, but this hardly alters the H^2^ scores even when assigning random classes to > 20% of the plant's pixels (see Additional file 2: Fig. S4), meaning this image assessment method is robust against segmentation errors.

**Fig. 8:**
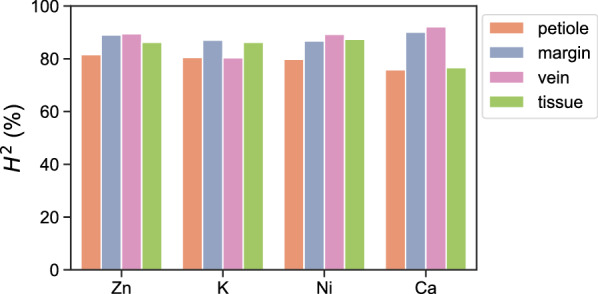
Broad-sense heritability (H^2^) of metal accumulation for each the four substructure CQ values for zinc (Zn), potassium (K), nickel (Ni), and calcium (Ca) for the petiole (orange), leaf margin (violet), leaf vasculature (vein; lilac) and leaf interveinal tissue (tissue; green) substructures

The H^2^ of substructure CQ is very high across all substructures and imaged metals, all above 75% (Fig. 8). In a way this is remarkable, as a pair-wise correlation between the metals shows that the CQs of substructures are only weakly correlated (See Additional file 2: Fig. S5), not exceeding r = 0.34. The only exceptions are the correlation between Ni and Ca CQ for the vasculature (*r* = − 0.55), and of Zn and Ni CQs (r ≥ 0.46 for any substructure). This means that while the H^2^ of substructure CQs are consistently high across metals, this is not caused by common biological processes, except perhaps for the regulation of Zn and Ni homeostasis in leaves.

### Heritability of metal colocalization

**Fig. 9:**
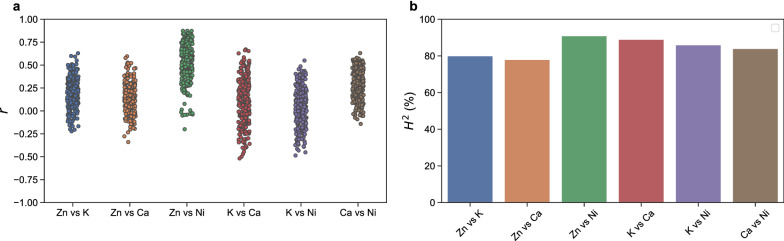
Colocalization of metals. **a** Correlation coefficients (*r*) for correlation of the zinc (Zn), calcium (Ca), potassium (K) and nickel (Ni) pixel-based concentration values per plant. **b** Broad-sense heritability (H^2^) of the correlation coefficients

While CQ could be used to quantify the spatial localization of metals, it does not quantify the colocalization of metal pairs. The extent of metal colocalization can be investigated by calculating a Pearson’s correlation coefficient per plant for the pixel values of every pair of metal images (Fig. 9). A remarkable diversity is observed with metal correlations ranging from weak negative correlations to moderately positive correlations depending on the accession. The exception is the correlation between Zn and Ni; for all plants there is a weak to strong positive correlation between Zn and Ni concentrations (Fig. 9a). The extent of colocalization between metal pairs varies between genotypes; plants within accessions display similar correlation coefficients, while correlation coefficient differences between accessions are relatively large. This results in high broad-sense heritability for all analysed metal pairs (Fig. 9b).

## Discussion

### Image-segmentation quality

For most leaf structures, the image segmentation was successful. Only image segmentation of the vasculature may need future improvement. While the segmentation of primary and secondary vasculature was successful, the prediction of tertiary vasculature was very unprecise. The current resolution of the µXRF image is lower than the width of most tertiary vasculature, resulting in the pixels only partially representing the reflectance of the vasculature, with a large influence of the interveinal tissue. This makes it difficult to accurately classify pixels as either vasculature or interveinal tissue. The reported segmentation accuracy is also influenced by errors in the manual ground-truth labelling. The problem of low resolution of the tertiary vasculature also challenges manual classification. The exact pixel at which the blade-edge starts is somewhat arbitrary, leading to some errors in setting the ground-truth for the margin segmentation. Similarly, the boundary between the substructures petiole and leaf margin is somewhat arbitrary to draw. Furthermore, in many images, a narrow strip of leaf blade can be seen running along the petiole, this cause misclassification of some leaf margin areas as petiole.

While a higher-resolution image could solve some of these problems, adjustment of the segmentation strategy could provide an alternative solution. Instead of classifying all pixels in the image, some regions of high uncertainty could be excluded. In particular, the tertiary vasculature and surrounding areas as well as the region where blade and petiole meet could be excluded. This approach was not used in the current investigation as the CQ-based H^2^ seemed robust enough against misclassification. Moreover, excluding specific regions could introduce some bias in the concentration quotients.

### Image segmentation sensitivity & generalizability

As expected, the segmentation method is sensitive to variation of the image processing parameters. While the general strategy of segmenting plant structures based on shape- and pixel intensity-attributes can be translated to other datasets and species, the exact algorithm and parameter values will need to be optimized for new settings. A custom segmentation algorithm would need to be created for every new species. When generalization to many other species is desired, we recommend the introduction of a machine-learning approach. To this end, the image processing strategy described in this article could be used to generate a labelled ground-truth dataset for multiple species. Next, a deep convolutional neural network could be trained to learn generalized substructure segmentation from this data [39]. Alternatively, synthetic data could be created by generating images based on structural plant models [40–42]. However, structural plant models would require reparameterization for individual species as well. This is likely to take an equal or longer time than the image processing strategy described here. It will however result in a significantly larger dataset.

### Image segmentation robustness

In many cases, the accuracy of image segmentation is essential for the reliable extraction of spatial attributes from metallome maps. However, because the calculated CQs are averaged over the plant substructures, the metric turned out to be robust against misclassification of plant pixels; insertion of noise in the segmentation algorithm did not affect CQ heritability when 20% of pixels were randomly classified. Remarkably, when noise is injected by assigning a random class to ≥ 50% of the pixels, heritability is still high. This is probably because the majority of the plant surface is taken up by the interveinal tissue. As the majority of pixels in each plant belongs to this interveinal tissue class, when random classes are assigned to a progressive percentage of pixels, the true underlying class becomes progressively dominated by the interveinal tissue class. Therefore, for injection percentages larger than 20%, the substructure CQs increasingly reflect the tissue CQ, which on its own has high H^2^ (see Additional file 2: Fig. S4).

### Heritability of metal accumulation patterns

Previous work linking phenotype to genotype through heritability has relied much on one-dimensional phenotypic traits such as yield components [43, 44], morphology [45], or photosynthetic efficiency [46, 47] and/or traits relating to plant geometry [47, 48]. Here, we translate spatial concentration patterns into a numerical metric to link them to genetic variability. This may allow for the discovery of novel genes and regulatory elements of these complex processes. In addition to the already established link of total metal accumulation to genotype [10], we show here that the spatial accumulation patterns are also heritable. The concentration quotient is a robust metric to determine heritability of metal accumulation patterns. We expect that this metric can be combined with data on single nucleotide polymorphisms between accessions to find novel genes and regulatory processes involved in metal homeostasis. The heritability of metal CQs is remarkably similar across different metals even though CQs for the different metals are only slightly correlated to one another. Some of the similarity in heritability across metals may be explained by the method with which µXRF intensity rasters were converted into concentration rasters; an equal thickness was assumed for the whole plant. If the thickness of the substructures varies individually between accessions, this may have affected the reported concentrations in the substructures. Therefore, some of the CQ heritability may relate to substructure thickness instead of metal accumulation. To counter this, any strong relationship between sample thickness and metal concentration would have shown up as a correlation between the pixel intensities of the different metals. This correlation was not observed. Still, for future investigations we recommend an estimation of the thickness of substructures [49]. No other phenotypic traits were found that explain the consistent patterns of substructure CQ heritability.

### The impact of the plantlet thickness on metal accumulation analysis through µXRF scanning

The thickness of *N. caerulescens* leaves varies, with petioles thicker than the foliar vasculature and the leaf lamina the thinnest. This may affect the calculation of total metal content based on µXRF scanning images. When we perform a sensitivity analysis for sample thickness using a portable XRF scanner, it is apparent that sample thickness variations between 200 and 800 μm (the thickness range observed for *N. caerulescens*) do not have a major effect on the intensity of the K and Ca signals. However, for the heavier Ni and Zn elements the difference becomes more pronounced (see Additional file 2: Fig S6). This will enhance any positive correlation of observed Zn and Ni concentrations but will only have a minor effect on the CQ values for these metals.

### Metal accumulation and colocalization patterns

While the biological interpretation of the images and the heritability analysis is not the focus of this report, and has not been analysed in great detail, it is still interesting to draw a few conclusions. *N. caerulescens* is a metal hyperaccumulator of three, perhaps even four, metals, Zn, Ni, Cd and Pb, which in itself is a rare trait. Zn and Ni hyperaccumulation is so far only described for the *Noccaea* genus and the *Dichapetalum* genus [50]. The few species in which natural Ni-Zn hyperaccumulation may occur are therefore likely to be members of the *Noccaea* genus. However, since high concentrations of both Ni and Zn in soil rarely occurs in nature, co-hyperaccumulation of both metals will only accidently occur in natural populations of *Noccaea* species. For all *N. caerulescens* plants we examined, wide ranges of colocalization were observed for the metal pairs, from no obvious colocalization to strong positive or negative colocalization. The only metal pair that showed consistent positive correlation in spatial concentrations was that of Zn and Ni. As noted above, this correlation may have been emphasized by local differences in leaf thickness that influence especially the Zn and Ni measurements but not those of Ca and K.

The positive colocalization we find appears to contrast with previous observations for *N. caerulescens* [16] and our earlier observations in the closely related *Noccaea tymphaea *[19] in which different localization patterns for Zn and Ni were found. One obvious difference in methodology between these reports and our work is that we exposed plants to both elevated Zn and Ni supply, in a 1:5 concentration ratio, to ensure accumulation of both metals. The work on *N. tymphaea* was performed on wild-collected plants and the previous work on *N. caerulescens* was performed on plants supplied with high Zn or with Ni supply, but not combined in one treatment. The extent of colocalization in *N. caerulescens* we studied, seems influenced by the genotype of the plant. In our analysis, we find accumulation of metals to associate largely within the vasculature, which is in line with previous findings for *N. caerulescens* in which Zn was localized using transverse leaf sections [18]. In the other reports on *N. tymphaea* and *N. caerulescens*, the highest Zn concentrations are found in the tissues surrounding the vasculature [16, 19], so slightly out of the vasculature. The aforementioned investigations consider only one or a few accessions. Since metal accumulation patterns are shown to be highly heritable, considering one accession will not be sufficient to make general claims about accumulation patterns on the species level.

## Conclusions

A general strategy for plant substructure segmentation of µXRF scans is proposed for plants similar to *N. caerulescens* in plant architecture, and a metric is developed to link phenotypic variance in the spatial distribution of the plant metallome to genetic variance. Spatial metal-accumulation patterns, as measured by CQ, are highly heritable in *N. caerulescens* for all metals and all segmented substructures. While image segmentation quality can benefit from higher resolution images, the segmentation quality was sufficient to extract highly heritable phenotypic traits on metal accumulation. Segmentation quality was highest for petiole and margin, and lowest for vasculature and leaf tissue. The quality depends on the image-processing parameters, which were optimized for the given dataset. The heritability scores for the various tested phenotypic traits were not significantly influenced by the segmentation quality when noise was injected. This indicates that the CQ-metric is robust to, at least some, segmentation error. The strategy employed in this study can be used to quantify elemental distributions patterns obtained from synchrotron or laboratory µXRF datasets of (hyperaccumulator) plants to assist in the comparative analysis of differential accessions, mutants or dose treatments, and thus be a valuable tool for the discovery of new genes that regulate metal homeostasis.

## Methods

### Plant material and cultivation

A diversity panel of 96 *N. caerulescens* accessions is used (Additional file 1), which have all been propagated for at least three generations by single-seed descent upon self-pollination to create phenotypically homogeneous lines. Before germination, surface-sterilized seeds are sown on 0.6-ml PCR-tubes of which the bottom is cut off and which are filled with 0.3% gelling agent (Gelzan, Sigma-Aldrich) (made with 0.5 × strength Hoagland’s nutrient solution). Seeds are stratified for six days at 3 °C, and a drop of 10 µM gibberellic acid (GA4 + 7; Duchefa) is applied on top to break any seed dormancy. After germination at 26 °C, tubes with germinated seeds are transferred to the hydroponics culture after three days, when the fully expanded cotyledons have emerged. The hydroponic system is set up in a temperature-controlled growth chamber. Four separate rectangular containers (11 cm height × 30 cm width × 40 cm length; 11 L each) are filled with a modified 0.5 × strength Hoagland’s nutrient solution: 3 mM KNO_3_, 2 mM Ca(NO_3_)_2_·4 H_2_O, 1 mM NH_4_H_2_PO_4_, 0.5 mM MgSO_4_·7 H_2_O, 1 µM KCl, 25 µM H_3_BO_3_, 2 µM MnSO_4_·4 H_2_O, 2 µM ZnSO_4_·7 H_2_O, 0.1 µM CuSO_4_·5 H_2_O, 0.1 µM as Na_2_MoO_4_·2 H_2_O and 20 µM Fe-HBED. The solution is set to pH 5.5 with KOH and buffered using 2 mM MES (2-(*N*-morpholino) ethane sulfonic acid). The solution is kept constantly aerated using an air stone at the bottom of each container. In each container, 35 plants are grown in open 0.6-mL Gelzan-filled bottomless PCR tubes inserted into 3 cm round plastic baskets with a foam disk to allow the roots to be immersed in the nutrient solution. After one week, the nutrient solution is replaced with a fresh solution, containing 20 µM ZnSO_4_ and 100 µM NiSO_4_ to induce accumulation of both Zn and Ni [10]. To calculate free ionic activity of Zn and Ni a simulation has been performed using the software GEOCHEM-EZ, with 90.2% of Zn present as the free ion, and 92.2% of Ni present as the free ion, in both cases the remainder is complexed with NO_3_, SO_4_ and PO_4_, while 100% of Fe^3+^ is complexed with HBED. The nutrient solutions are changed completely after one week, at which interval the pH has not changed substantially (< 0.2 pH change). Plants are grown for 20 days in hydroponics with a 12–12 h light–dark photoperiod, under high intensity full spectrum Valoya B200 LED lights (Valoya Oy, Helsinki Finland), with a photosynthetic photon flux density at of 350 μmol m^2^ s^−1^ (measured with an Apogee MQ-500 instrument) at 26/20 °C day/night temperatures.

### Elemental mapping using µXRF

Before being destroyed by acid digestion, freshly hydrated, plantlets of which the roots were cut off, were mounted and scanned using a modified ATLAS X instrument (IXRF Systems) at the Centre for Microscopy and Microanalysis, The University of Queensland, Australia. On the µXRF motion stage (300 × 300 mm), the whole plantlets were mounted, and to retain their position, shape, and moisture content during the measurement, the plantlets were sandwiched between a 100-μm thick cellulose acetate sheet (below) and a sheet of 6-μm thick Ultralene thin film (top). Prior to the mounting of the plantlets the main root was cut off and thereafter the whole plantlet was tightly sealed in the mounting with thin film. There was no visible leakage from the cut surface. The µXRF system is equipped with two 50-W X-ray sources fitted with polycapillary focussing optics: XOS microfocus Mo-target tube producing 17.4 keV X-rays (flux of 2.2 × 10^8^ ph s^−1^) focussing to 25 μm FWM and a Rh-target tube producing 20.2 keV (flux of 1.0 × 10^7^ ph s^−1^) focussing to 5 μm FWM. It has two KETEK H150 silicon drift detectors of 150 mm^2^ coupled to a XIA Mercury X4 signal-processing unit. Typical energy resolution is < 145 eV with a maximum input count rate of 2 M counts per second. During measurements of all plantlets, all parameter settings and environments were kept constant; temperature at ~ 20 °C, using the Mo 25 µm X-ray source at a 50 kV, 1000 µA, a rise time of 0.25 µs and a per-pixel dwell of 100 ms. The XRF spectra on the UQ µXRF facility were acquired in mapping mode using the instrument control package (Iridium, IXRF Systems) from the sum of counts at the position of the principal K-line fluorescence peak for each element. These were each exported into ImageJ as greyscale 8-bit TIFF files. The sum of counts images for Zn, K, Ca, and Ni as well as the Compton scatter image were used here.

### Chemical analysis of plant samples

At the end of the cultivation period, the plants were harvested and divided into shoots and roots. While the shoots were mounted for µXRF scanning (see above), the root samples were rinsed with de-ionised water and dried at 60 °C for 72 h in a dehydrating oven. After scanning, also the shoot samples were collected from the cellulose acetate sheet and oven dried. Each sample was then weighed (~ 50 mg) and digested using 1 mL HNO_3_ (70%) in a hot block (ThermoFisher Digital Dry Bath) with a 1-h programme at 70 °C and a further  1-h at 125 °C, and then diluted to 10 mL with ultrapure water (Millipore 18.2 MΩ·cm at 25 °C) for subsequent analysis. Aliquots were analysed by Inductively Coupled Plasma Atomic Emission Spectroscopy (ICP-AES, Thermo Scientific iCAP 7400) for the macro-elements Na, Mg, Al, P, S, K and Ca and the trace elements Mn, Fe, Cu, Ni and Zn in radial and axial modes depending on the element and expected analyte concentration. All elements were calibrated with a four-point curve covering analyte ranges in the samples. In-line internal addition standardization with yttrium was used to compensate for matrix-based interferences. Quality controls included matrix blanks, certified reference material (Sigma-Aldrich Periodic Table Mix 1 for ICP TraceCERT®, 33 elements, 10 mg L^−1^ in HNO_3_), and Standard Reference Material (NIST Apple 1515 digested with HNO_3_).

### Segmentation of plant substructures

Substructure classes were assigned to every pixel in the µXRF scans. One pixel can belong to one of the following five classes: background, petiole, leaf margin (margin), vasculature (vein) or leaf interveinal tissue (tissue). Several traditional image processing techniques were used in sequence to segment the substructures (Fig. 2).

To create the plant foreground mask, the K, Ca, Ni and Zn grayscale images were binarized using the Otsu thresholding method, which finds the intensity threshold that separates the pixel intensities in two classes based on a minimization of the intra-class intensity variance [51]. This results in four binary masks for each metal type; ${M}_{\text{K}}$, ${M}_{\text{Ca}}$, ${M}_{\text{Ni}}$, and ${M}_{\text{Zn}}$. The union of these masks resulted in the plant-foreground mask:

1 $${M}_{\text{plant}}={M}_{\text{K}}\cup {M}_{\text{Ca} }\cup {M}_{\text{Ni}}\cup {M}_{\text{Zn}}$$

Using morphological image processing [52], the foreground mask could be divided into a blade and a petiole based on the feature that petioles are less wide than blades. An opening operation with a structuring element of size 15 × 15 was applied in a single iteration to remove the petiole and yield the blade mask.

2 $${M}_{\text{blade}}=\text{opening}({M}_{\text{plant}},15\times 15)$$

Subtracting the blade mask from the plant mask provided the petiole mask. To remove small artefacts from the petiole mask, a connected-component analysis was done and all connected components with an area smaller than 0.001% of the image area were discarded:

3 $${M}_{\text{petiole}}={\text{coco}(M}_{\text{plant}}- {M}_{\text{blade}},0.001\%)$$

A mask of the leaf margin with a width of three pixels (corresponding to ~ 100 µm) is obtained by taking the internal gradient, which is the difference between the blade mask and the erosion of the blade mask with a structuring element of size $3\times 3$:

4 $$M_{{{\text{margin}}}} = M_{{{\text{blade}}}} - M_{blade} { \ominus } b,$$

where ${ \ominus }$ is erosion and $b$ is the 5 × 5 structuring element.

The vein mask was created based on the Compton scatter image, ${I}_{\text{Compton}}$, which shows the vasculature with higher intensities compared to the leaf tissue. A Laplacian operator with a kernel size of 7 × 7 was used to calculate the second-order derivative of the Compton scatter image in order to detect. The Laplacian highlights regions in the image with rapid intensity change, thus highlighting the vein structure. The result of the Laplacian operation was used in two ways. The thicker primary and secondary vasculature were segmented by thresholding the Laplacian image ${M}_{\text{vein}1}$, and the thin tertiary vasculature was located by thresholding the Laplacian image for values below 0 followed by a skeletonization to reduce the thickness of the tertiary vasculature ${M}_{\text{vein}2}$. The combined vein mask was obtained from ${M}_{\text{vein}1}{M}_{\text{vein}2}$ and an intersection to exclude detections on the leaf margin:

5 $${M}_{\text{vein}1}={\text{Laplacian}(I}_{\text{Compton}})<-2500$$

6 $${M}_{\text{vein}2}={\text{skeleton}(\text{Laplacian}(I}_{\text{Compton}})<0)$$

7 $${M}_{\text{vein}}={(M}_{\text{vein}1}\cup {M}_{\text{vein}2}) \cap ( {M}_{\text{blade}}- {M}_{\text{margin}})$$

Finally, the leaf-tissue mask was obtained by taking the difference between the blade mask, the margin mask, and the vein mask.

8 $${M}_{\text{tissue}}={M}_{\text{blade}}- {M}_{\text{margin}}- {M}_{\text{vein}}$$

With the four masks, ${M}_{\text{blade}}$, ${M}_{\text{margin}}$, ${M}_{\text{vein}}$, and ${M}_{\text{tissue}}$, every pixel in the image is classified in one of the four substructure classes. All image processing steps were carried out using the NumPy (1.19.2) and OpenCV-Python (4.2.0.34) packages for Python.

### Evaluation of segmentation

To determine the segmentation quality, for each of the petiole, margin, vein, and tissue substructure classes, 1000 predicted pixels were randomly selected and displayed to a researcher unaware of their predicted class. These pixels were then manually classified as belonging to one of the four classes to obtain a ground-truth classification and to calculate the precision, recall and F1-scores per class: A sensitivity analysis of the F1-score was run on four parameters of the segmentation algorithm.

9 $${\text{Precision}}_{i}= \frac{{M}_{ii}}{{\sum }_{j}{M}_{ji}}$$

10 $${\text{Recall}}_{i}= \frac{{M}_{ii}}{{\sum }_{j}{M}_{ij}}$$

11 $${\text{F}1}_{i}= 2*\frac{{\text{Precision}}_{i}*{\text{Recall}}_{i}}{{\text{Precision}}_{i}+{\text{Recall}}_{i}}$$

where $M$ is the confusion matrix that holds the ground-truth classes in the rows and the predicted classes in the columns. ${M}_{rc}$ indicates the value in the confusion matrix on row $r$ and column $c$, which indicates how often a true class $r$ was predicted as class $c$.

A sensitivity analysis of the F1-score was run on four parameters of the segmentation algorithm. Parameter values were incremented from two steps below to two steps above the chosen parameter values. The structuring element used for the opening operation that yielded the blade mask ${M}_{\text{blade}}$ was varied from 7 × 7 to 23 × 23. The Laplacian operator kernel was varied from 3 × 3 to 11 × 11. The threshold on ${\text{Laplacian}(I}_{\text{Compton}})$ that yielded ${M}_{\text{vein}1}$ (Eq. 5) was varied from − 3500 to − 1500. The threshold on ${\text{Laplacian}(I}_{\text{Compton}})$ that yielded ${M}_{\text{vein}2}$ (Eq. 6) was varied from − 200 to 200.

### Random substructures

In addition to the segmented substructures, “random substructures” were generated. A random mask was created to compare heritability of phenotypical traits for real substructures (petiole, margin, tissue, veins) to a random non-existing substructure. The heritability in these non-existing substructures was expected to be low and served as a negative control of our heritability calculations.

For every plant, a random mask was generated consisting of 5 squares of 30 × 30 pixels at a random location on the plant surface. Where these squares overlapped with the background, they were cropped to be fully contained within the boundaries of the plant mask.

12 $${M}_{r\text{andom}}=\text{random}\_\text{squares}\left({M}_{\text{plant}}\right)\cap {M}_{\text{plant}}$$

### Concentration Quotient

The images of metal concentrations can be translated into a numerical trait by analysing the accumulation in plant substructures relative to the whole plant. To this end we introduce the “concentration quotient” (CQ) as the mean concentration in a plant substructure (e.g., the petiole) divided by the mean concentration in the whole plant:

13 $$\text{CQ}= \frac{\frac{1}{{N}_{s}} \sum_{(x,y)\in {A}_{s}}(P\left(x,y\right))}{\frac{1}{{N}_{p}} \sum_{(x,y)\in {A}_{p}}(P\left(x,y\right))}[-]$$

where ${N}_{p}$ and ${N}_{s}$ are the number of pixels belonging to respectively the plant and the substructure,${A}_{p}$ and ${A}_{s}$ are the set of pixels belonging respectively to the plant and to a substructure, and $P\left(x,y\right)$ is the µXRF derived concentration of a pixel at location $\left(x,y\right)$. If $\text{CQ}=1$, the mean concentration within a substructure is equal to that of the plant. $\text{CQ}>1$ shows relative higher concentration within the substructure, while $\text{CQ}<1$ shows relative lower concentration within a substructure. CQ allows for an estimation of the heritability of spatial metal distribution.

### Heritability and other statistics

The contribution of genotype to the observed phenotypes was calculated by estimating the broad-sense heritability (H^2^, also called repeatability [53]):

14 $${H}^{2}=\frac{Var(G)}{Var(T)}$$

$Var(G)$ is the variance of the genotype and $Var(T)$ is the total variance. The H^2^ scores were calculated for the CQs of Zn, K, Ca, and Ni for the petiole, leaf margin, leaf interveinal tissue and leaf vasculature substructures (in total 16 traits). For comparison, H^2^ was also calculated for plant size and the mean plant concentration of the four metals. Variance components were estimated using mixed models of the following form:

15 $$P=G+ \varepsilon$$

where both G (genotype) and $\varepsilon$ (error) are random terms in the mixed model. A mixed model is fit so that the *n* accessions are represented by a vector β of size *n* that estimates the observed numerical phenotypes of the replicates as closely as possible. The difference between this estimation and the actual observed numerical phenotype results in the error term $\varepsilon .$ $Var(G)$ is the variance of β. $Var(T)$ is the sum of $Var(e)$ and $Var(G)$.

16 $$Var\left(T\right)=Var\left(G\right)+Var(\varepsilon )$$

All models were checked for assumptions of normality and equal variance. Assumptions hold for all models except those in which the CQ of a random substructure was used as phenotype (see “random substructures” below). In these cases, there is some correlation between fitted model values and model residuals. The variance components were estimated by the VarCorr function from the lme4 package in R statistical software. Total variance was calculated as the sum of the $Genotype$ and $\varepsilon$ variance.

All reported correlation coefficients (*r*) were calculated using Pearson’s correlation coefficient. Code is available at https://github.com/LucasYEAST/noccaea.

### Sensitivity analysis on the effect of the sample thickness

The thickness of the imaged rosette leaves is not constant. To assess the impact of sample thickness on the accuracy of metal concentration calculations, seventeen leaves of N. *caerulescens* were used to cut 6-mm diameter discs from, that were dried in the oven for 48 h at 60 °C. The disc leaves were measured using a portable XRF instrument (Thermo Fisher Scientific Niton XL3t 950 GOLDD+) on top of a sheet of paper, a 2-mm thick titanium (99.995% pure) plate, and a 2-mm thick molybdenum (99.995% pure) plate [49], followed by acid digestion for ICP-AES analysis. The spectra of the disc leaves were processed using the GeoPIXE software (http://nmp.csiro.au/GeoPIXE.html), and the modelled thickness parameter was adjusted to vary from 200 µm to 800 µm with an increment of 100 µm. The concentrations determined by ICP-AES analysis were used as a comparison with concentrations calculated based on GeoPIXE analysis.

## Supplementary Information

**Additional file 1.** List of *Noccaea caerulescens* accessions used.

**Additional file 2: Figure S1.** Scatter plots showing μXRF- versus ICP-AES determined total metal content data per plant. **Figure S2.** Sensitivity analysis on recall and precision. a), e) kernel size used for morphological opening operation on binary plant masks to yield blade and petiole separation. b), f) kernel size of Laplacian operator to detect edges. c), g) Binary threshold on the calculated Laplacian to segment thinnest vasculature d), h) Binary threshold on the calculated Laplacian to segment wider vasculature. Values on the X-axis with asterisk are used for all analyses. **Figure S3.** Correlation coefficients for pairwise correlations of the four substructure CQ for the four metals investigated. **Figure S4.** Robustness of CQ to incorporation of noise. a,b,c,d,e,f show the distribution of actual pixel classes under the noise-injected masks. For noise injection > 20% all classes in the noise masked have a majority of pixels that actually belong to the “tissue”-class. g) Broad-sense heritability (H^2^) of zinc CQ for the four substructures where the classification of substructures has been injected with increasing amounts of class noise. Percentages denote the fraction of pixels in the plant that has been assigned a random substructure class. Injection of noise does not decrease H^2^. **Figure S5.** Correlation coefficients for correlations between the CQs of metal-pairs for all substructures. **Figure S6.** The ratios of metal concentrations as calculated based on GeoPIXE analysis of portable XRF data, compared to the concentrations determined by ICP-AES, as a function of the sample thickness parameter set in the GeoPIXE quantification. Ni concentrations are below the detection limit of the portable XRF instrument and are not included. **Table S1.** Correlation of substructure CQ with three other plant traits.

## Data Availability

The code created during the current study is available in the Noccaea repository, https://github.com/LucasYEAST/noccaea. The datasets used and/or analysed during the current study are available from the corresponding author on reasonable request.

## Abbreviations

µXRF: Micro-X-ray fluorescence microscopy
CQ: Concentration quotient
H^2^: Broad-sense heritability
XRF: X-ray fluorescence microscopy
margin: Leaf margin
vein: Leaf vasculature
tissue: Leaf interveinal tissue
