# Supplementary material for: Quantification of spatial metal accumulation patterns in Noccaea caerulescens by X-ray fluorescence image processing for genetic studies
Source: Plant Methods. 2021 Aug 3;17:86. doi: 10.1186/s13007-021-00784-9 (PMC8336263; doi:10.1186/s13007-021-00784-9)
Supplement: Supplementary file 2 — Additional file 2: Figure S1. Scatter plots showing μXRF- versus ICP-AES determined total metal content data per plant. Figure S2. Sensitivity analysis on recall and precision. a), e) kernel size used for morphological opening operation on binary plant masks to yield blade and petiole separation. b), f) kernel size of Laplacian operator to detect edges. c), g) Binary threshold on the calculated Laplacian to segment thinnest vasculature d), h) Binary threshold on the calculated Laplacian to segment wider vasculature. Values on the X-axis with asterisk are used for all analyses. Figure S3. Correlation coefficients for pairwise correlations of the four substructure CQ for the four metals investigated. Figure S4. Robustness of CQ to incorporation of noise. a,b,c,d,e,f show the distribution of actual pixel classes under the noise-injected masks. For noise injection > 20% all classes in the noise masked have a majority of pixels that actually belong to the “tissue”-class. g) Broad-sense heritability (H2) of zinc CQ for the four substructures where the classification of substructures has been injected with increasing amounts of class noise. Percentages denote the fraction of pixels in the plant that has been assigned a random substructure class. Injection of noise does not decrease H2. Figure S5. Correlation coefficients for correlations between the CQs of metal-pairs for all substructures. Figure S6. The ratios of metal concentrations as calculated based on GeoPIXE analysis of portable XRF data, compared to the concentrations determined by ICP-AES, as a function of the sample thickness parameter set in the GeoPIXE quantification. Ni concentrations are below the detection limit of the portable XRF instrument and are not included. Table S1. Correlation of substructure CQ with three other plant traits. [file 13007_2021_784_MOESM2_ESM.docx]

**Heritability analysis of spatial metal accumulation patterns in**

***Noccaea caerulescens* by X-ray fluorescence image processing**

**Additional file**

*Lucas van der Zee^1^, Amelia Corzo Remigio^3^, Lachlan W. Casey^4^, Imam Purwadi^3^, Jitpanu Yamjabok^2^, Antony van der Ent^3^, Gert Kootstra,^1*^ Mark G. M. Aarts^2*^*

^1^Department of Plant Sciences, Farm Technology, Wageningen University and Research,

The Netherlands.

^2^Laboratory of Genetics, Wageningen University and Research, The Netherlands.

^3^Centre for Mined Land Rehabilitation, Sustainable Minerals Institute,

The University of Queensland, Australia.

^4^Centre for Microscopy and Microanalysis, The University of Queensland, Australia.

*Corresponding author: mark.aarts@wur.nl

# Additional figures


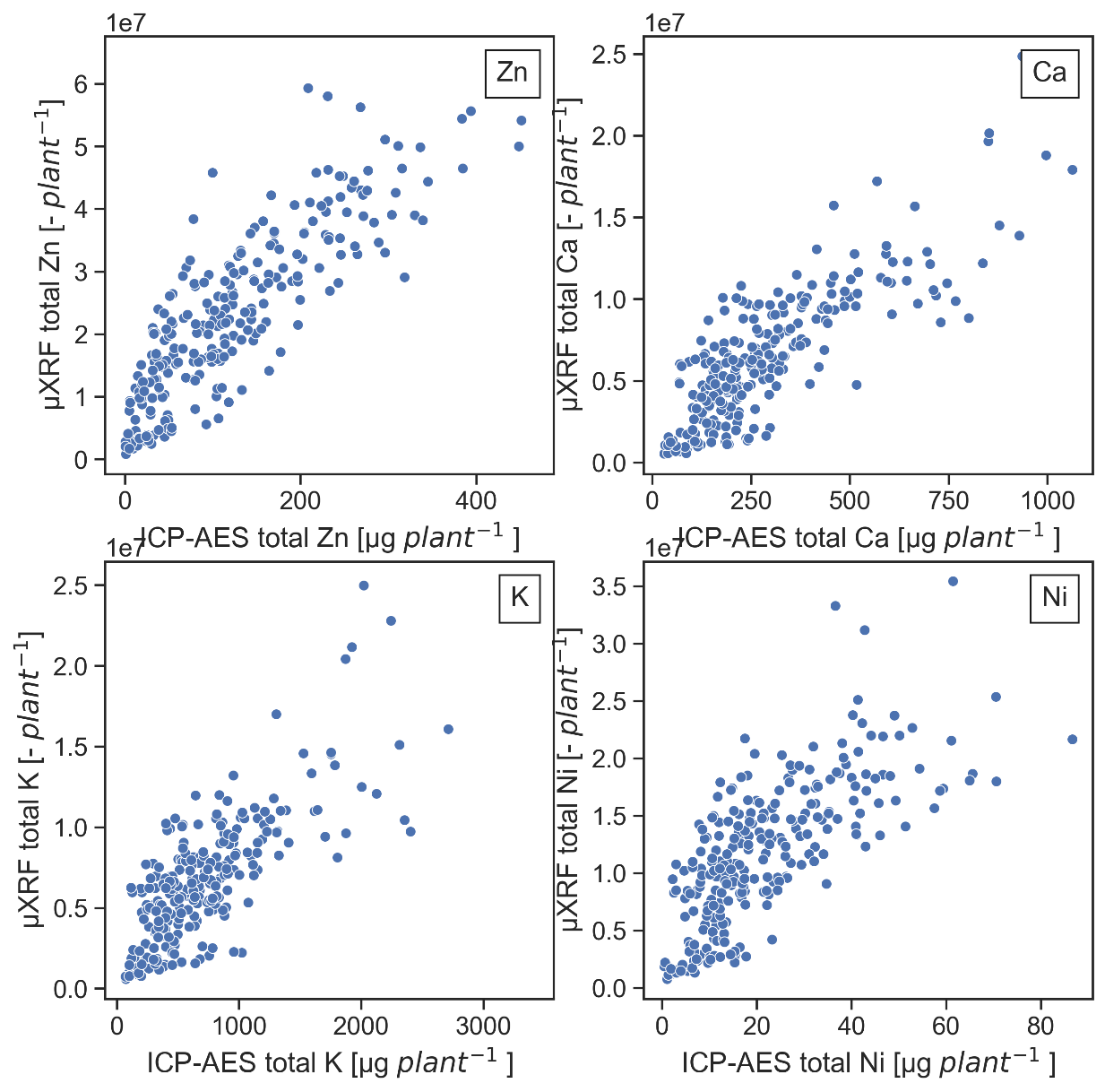


*Figure S1:* Scatter plots showing μXRF- versus ICP-AES determined total metal content data per plant.


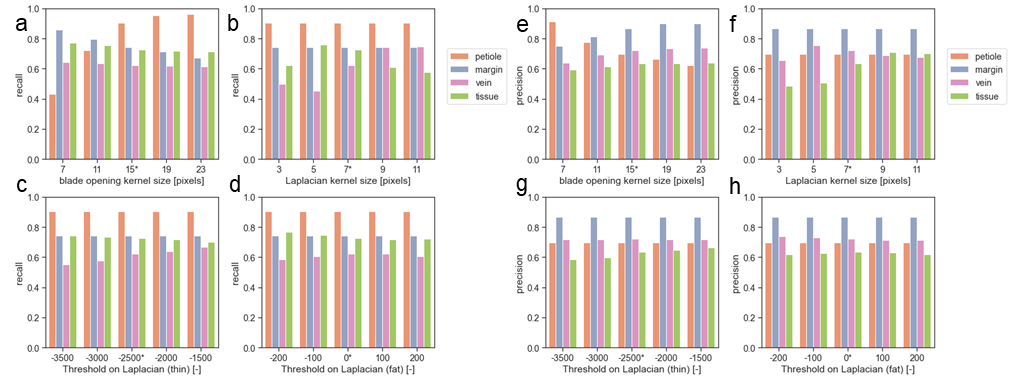


*S2: Sensitivity analysis on recall and precision. a), e) kernel size used for morphological opening operation on binary plant masks to yield blade and petiole separation. b), f) kernel size of Laplacian operator to detect edges. c), g) Binary threshold on the calculated Laplacian to segment thinnest vasculature d), h) Binary threshold on the calculated Laplacian to segment wider vasculature. Values on the X-axis with asterisk are used for all analyses.*


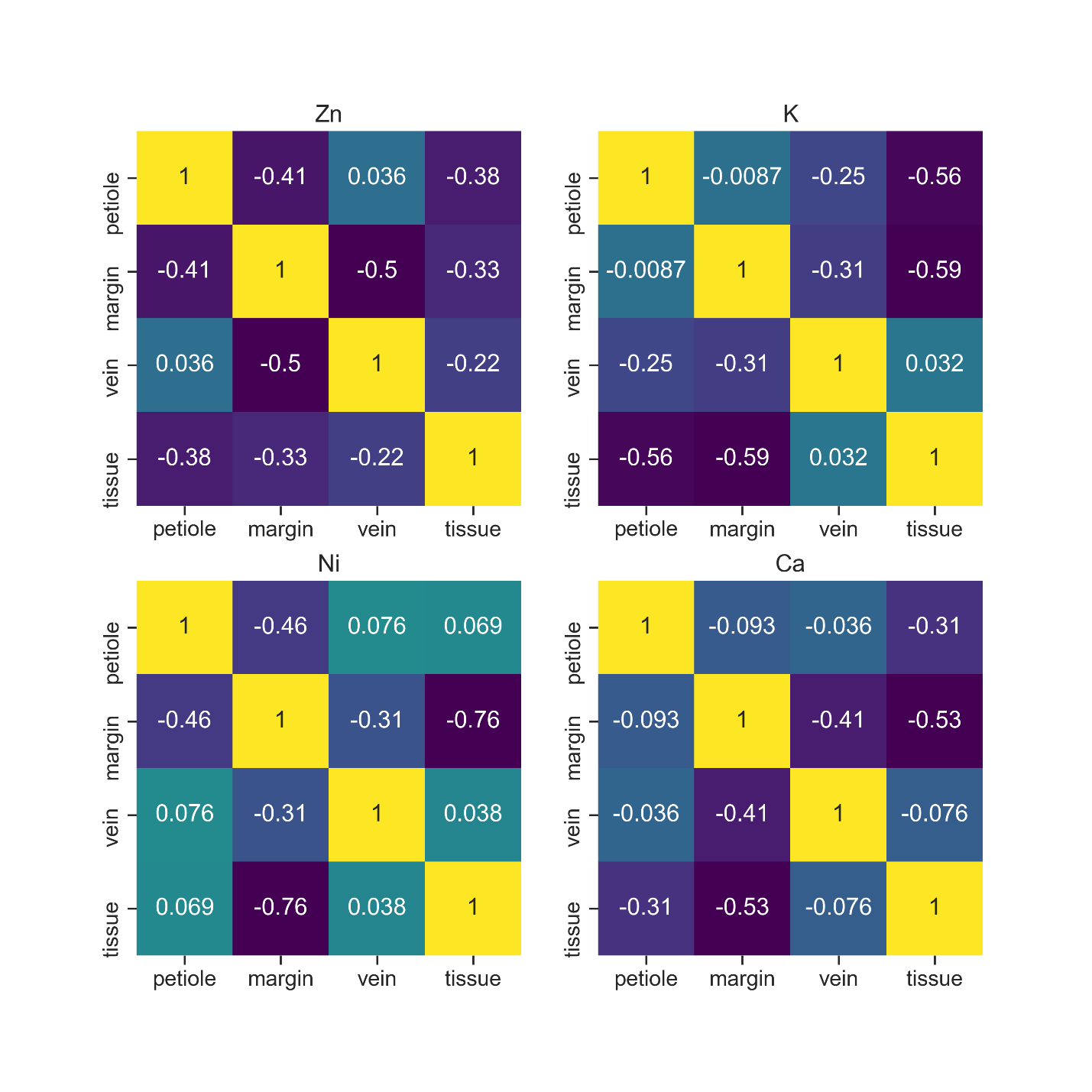


*Figure S3: Correlation coefficients for pairwise correlations of the four substructure CQ for the four metals investigated.*


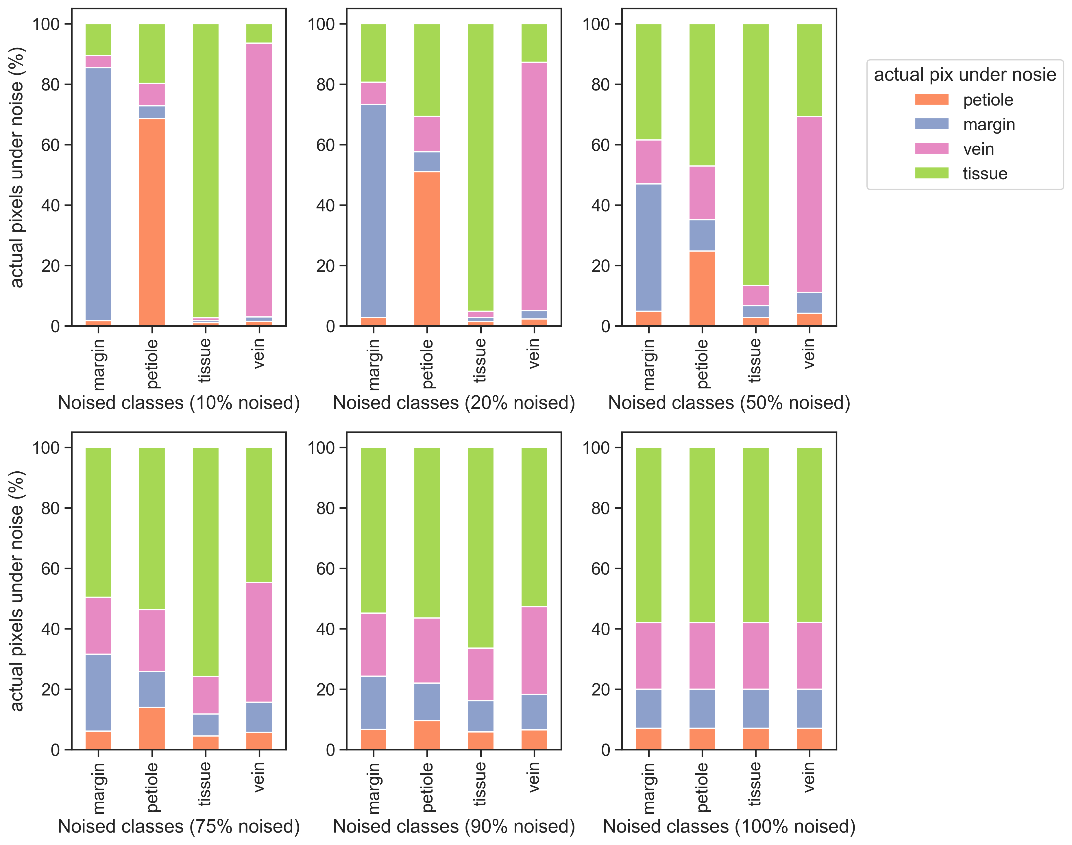

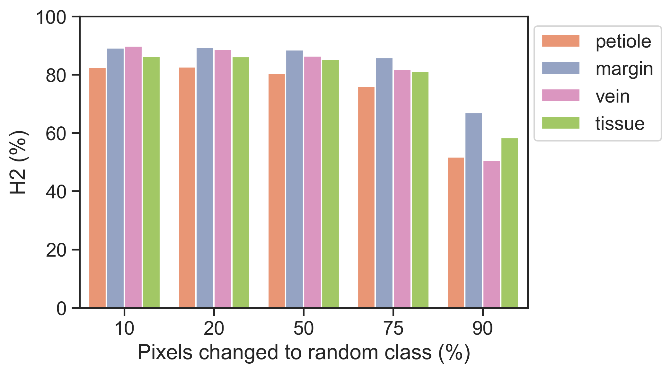


a

b

c

d

e

f

g

*Figure S4: Robustness of CQ to incorporation of noise. a,b,c,d,e,f show the distribution of actual pixel classes under the noise-injected masks. For noise injection >20% all classes in the noise masked have a majority of pixels that actually belong to the “tissue”-class. g) Broad-sense heritability (H^2^) of zinc CQ for the four substructures where the classification of substructures has been injected with increasing amounts of class noise. Percentages denote the fraction of pixels in the plant that has been assigned a random substructure class. Injection of noise does not decrease H^2^.*


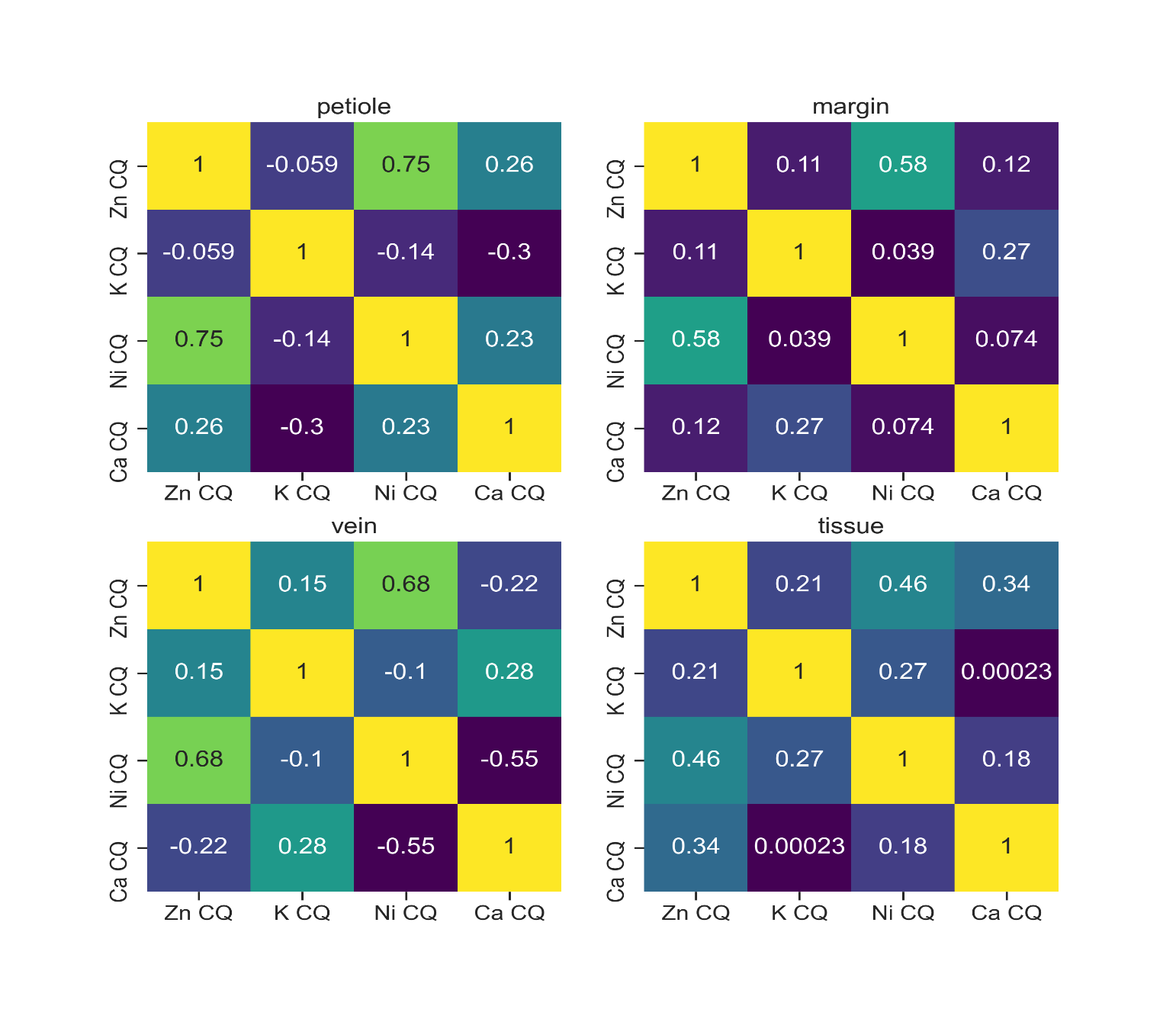


*Figure S5: Correlation coefficients for correlations between the CQs of metal-pairs for all substructures.*


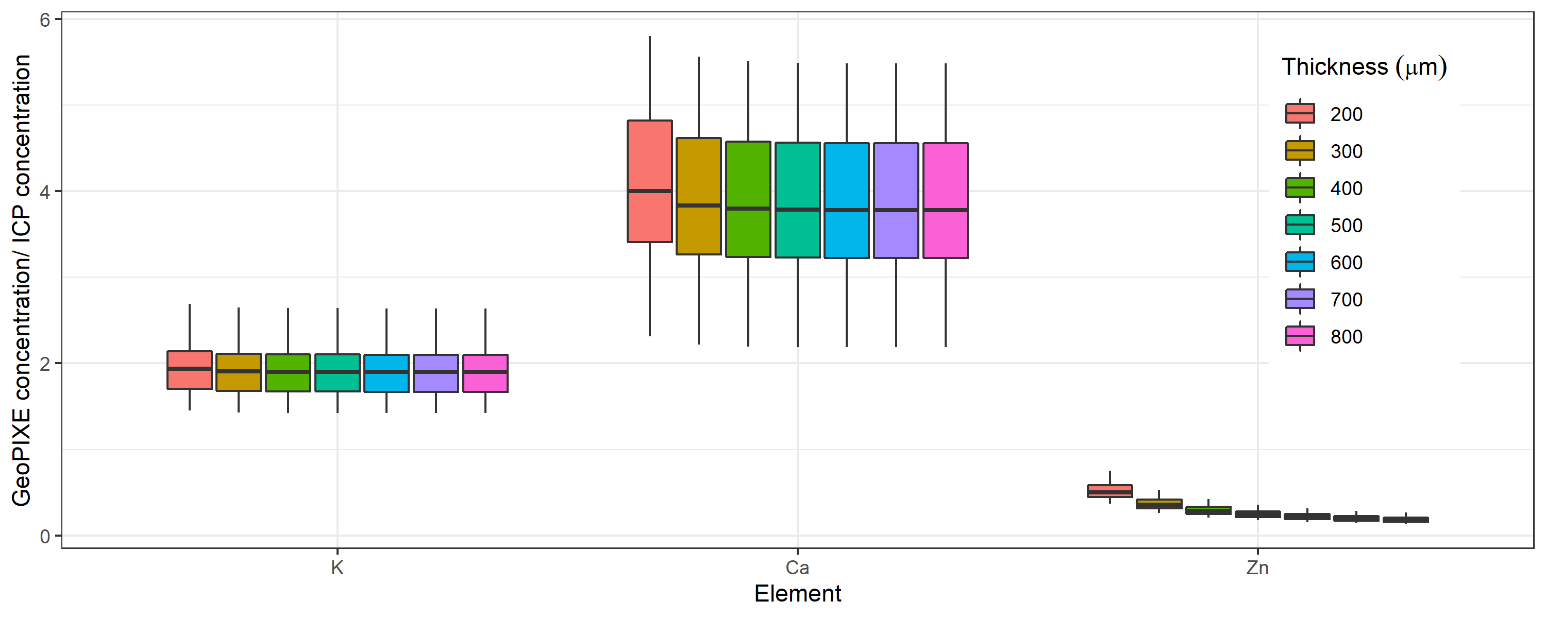
*Figure S6****.*** *The ratios of metal concentrations as calculated based on GeoPIXE analysis of portable XRF data, compared to the concentrations determined by ICP-AES, as a function of the sample thickness parameter set in the GeoPIXE quantification. Ni concentrations are below the detection limit of the portable XRF instrument and are not included.*

*Table S1: Correlation of substructure CQ with three other plant traits.*

| Metal | substructure | plant size *r* | mean metal *r* concentration *r* | substructure fractional area *r* |
| --- | --- | --- | --- | --- |
| Zn |  |  |  |  |
|  | petiole CQ | 0.09 | 0.04 | 0.05 |
|  | margin CQ | -0.02 | -0.03 | 0.12 |
|  | vein CQ | 0.15 | 0.41 | 0.31 |
|  | tissue CQ | -0.05 | -0.22 | 0.08 |
| K |  |  |  |  |
|  | petiole CQ | 0.05 | -0.06 | 0.04 |
|  | margin CQ | -0.04 | 0.33 | 0.15 |
|  | vein CQ | -0.24 | -0.34 | 0.15 |
|  | tissue CQ | -0.16 | -0.08 | 0.03 |
| Ni |  |  |  |  |
|  | petiole CQ | 0.10 | -0.09 | 0.08 |
|  | margin CQ | 0.01 | 0.22 | -0.01 |
|  | vein CQ | 0.41 | 0.38 | 0.29 |
|  | tissue CQ | 0.09 | -0.20 | 0.15 |
| Ca |  |  |  |  |
|  | petiole CQ | -0.15 | 0.00 | 0.14 |
|  | margin CQ | -0.01 | 0.10 | 0.00 |
|  | vein CQ | -0.55 | -0.55 | 0.01 |
|  | tissue CQ | 0.08 | 0.28 | 0.00 |
